# Stereoselective Promiscuous Reactions Catalyzed by Lipases

**DOI:** 10.3390/ijms23052675

**Published:** 2022-02-28

**Authors:** Angela Patti, Claudia Sanfilippo

**Affiliations:** CNR—Istituto di Chimica Biomolecolare, Via Paolo Gaifami 18, I-95126 Catania, Italy; claudia.sanfilippo@cnr.it

**Keywords:** lipases, stereoselectivity, promiscuous activity, C-C bond forming reactions

## Abstract

The ability of lipases to display activity beyond their physiological reactions, so-called “catalytic promiscuity”, has gained increasing interest in the last two decades as an important tool for expanding the application of these enzymes in organic synthesis. Some lipases have been shown to be effective in catalyzing a variety of C-C bond formation reactions and most of the investigations have been directed to the optimization of the products yield through a careful tuning of the experimental parameters. Despite the fact that new stereogenic carbons are formed in many of the tested reactions, the target products have been often obtained in racemic form and examples of an efficient asymmetric induction by the used lipases are quite limited. The aim of this review, mainly focused on those lipase-catalyzed promiscuous reactions in which optically active products have been obtained, is to offer a current state of art together with a perspective in this field of asymmetric synthesis.

## 1. Introduction

Biocatalysis is well recognized as an important tool in “green chemistry” thanks to the mild reaction conditions required and the excellent properties of chemo-, regio-, and stereoselectivity of the enzymes that contribute to drive the reaction outcome toward the synthesis of target products minimizing unwanted side-products with simplification of the purification steps [1]. The portfolio of available enzymes and biocatalyzed reactions applied in organic synthesis is continuously expanding, also with the aid of direct evolution techniques [2,3], and in many cases the optimization of enzymatic performances suitable for industrial applications has been achieved [4,5].

Furthermore, it has been observed that some enzymes display unexpected activity with respect their biological specificity, a phenomenon called “enzyme promiscuity” and sub-classified in a) condition promiscuity, related to the ability of an enzyme to work in non-physiological reaction conditions; b) substrate promiscuity, as the ability to recognize and transform unexpected substrates; c) catalytic promiscuity, referred to the ability to catalyze reactions other than those characteristic of their biological role [6]. Enzyme promiscuity has attracted great interest in the last decade as an emerging opportunity to further broaden the application of biocatalysis in organic synthesis and large investigations has been carried out on a variety of enzymes [7,8,9,10] as well as on the origin of promiscuity [11] 

In this context, lipases (triacylglycerol acylhydrolase, EC 3.1.1.3), which physiologically catalyze the hydrolysis of fats through the nucleophilic attack of water on an acyl-enzyme intermediate, occupy a place of honor for the displayed wide range of promiscuity. Their condition promiscuity has been known since late 1980s when their ability to work in organic solvents was evidenced by Klibanov’s group [12] and more recently extended to other non-conventional media as ionic liquids (ILs) [13] or deep eutectic solvents (DES) [14]. In these non-aqueous reaction media, the lipase activity can be driven toward reversed reactions to hydrolysis by replacing water with other nucleophiles, such as alcohols or amines. The substrate promiscuity of lipases is witnessed by the impressive molecular diversity of compounds that can be synthesized through lipase-catalyzed transesterification or aminolysis reactions, with concomitant kinetic resolution of racemic mixtures [15,16] or desymmetrization of *meso*- or prochiral substrates [17].

Furthermore, the immobilization of lipases on a variety of inert supports [18] provides additional advantages in facilitating the catalyst recovery and recycling, in increasing the enzyme stability and modulating the catalytic features, all these factors contributing to the development of industrial sustainable biotransformations [19]. 

Although the first carbon-carbon (C-C) bond forming reaction catalyzed by lipases was reported in 1986 [20], the study of catalytic promiscuity of lipases took off in the 2000s after Berglund and coworkers succeeded in inducing aldolase activity in a *Candida antarctica* B Ser105Ala mutant to obtain the aldol from self-condensation of hexanal [21]. Following this landmark paper, a variety of other promiscuous activities have been reported for lipases and recently reviewed [10]. 

As could be expected, for such novel enzymatic reactions, most investigations have been focused on maximizing the yield of the target products through a careful tuning of the experimental conditions. Less attention has been addressed to the stereoselectivity of these biotransformations, which in many cases lead to the formation of new stereogenic carbons. Although enzymatic nature of catalysis has been demonstrated, the formation of racemic compounds has been often reported and examples of an efficient asymmetric induction by lipases in promiscuous reactions are still limited.

In this review, the development of asymmetric lipase-catalyzed promiscuous reactions, classified by typology, is shortly outlined, limiting the discussion to those examples for which data on the stereoselectivity are available, with the aim to stimulate further investigation in chemistry as well as in protein biochemistry for a better understanding and engineering of the lipase selectivity. 

## 2. Lipase-Catalyzed Aldol Reactions

Aldol condensation between two carbonyl compounds is one of the most important C-C bond forming reaction, leading to the formation of β-hydroxy carbonyl compounds with simultaneous generation of one or two new stereocentres. Catalytic asymmetric aldol condensation has been widely investigated by using different approaches [22] and a variety of enantioselective catalysts has been developed. 

**Scheme 1:**
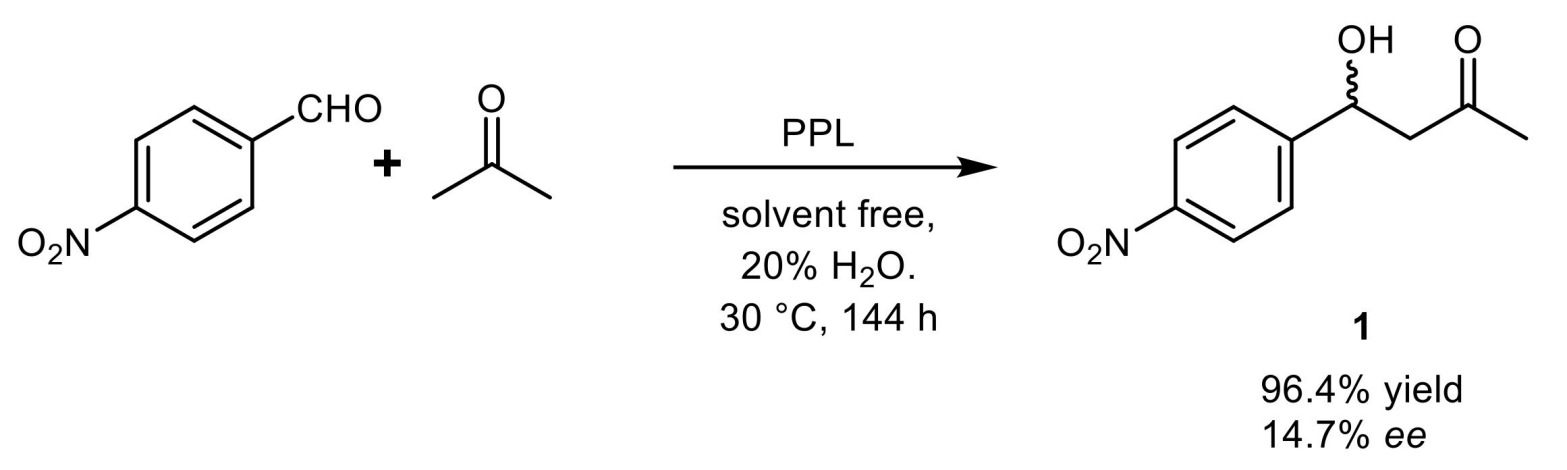
PPL-catalyzed aldol condensation of 4-nitrobenzaldehyde and acetone.

The first asymmetric lipase-catalyzed aldol condensation was reported in 2008 for 4-nitrobenzaldehyde and acetone in the presence of porcine pancreas lipase (PPL), that gave the best results among five tested enzymes, allowing to obtain 96.4% of substrate conversion over 144 h with low enantioselectivity (14.7% enantiomeric excess, *ee*) (Scheme 1). The water content greatly influenced the reaction rate, which reached a maximum at 20% concentration, whereas better stereoselectivity was observed in the presence of 1% of water (43.6% *ee*) at the expense of the product yield (11.7%) [23].

**Scheme 2:**
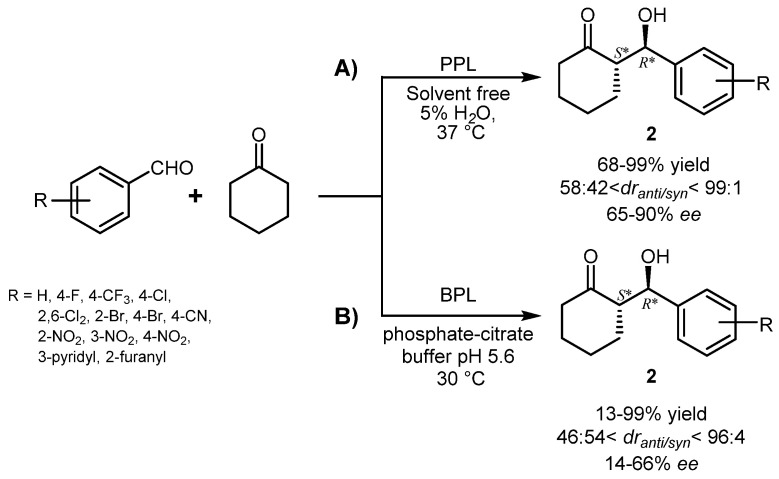
Aldol condensation of aromatic aldehydes and cyclic ketones catalyzed by (**A**) PPL or (**B**) BPL in different reaction media.

The same research group later investigated the reaction of different aromatic aldehydes with cyclic ketones and PPL showed the best catalytic performances in “wet” (5% of water) cyclohexanone as reaction medium giving the expected aldols **2** in 68–99% yield with *anti* stereopreference (diastereoisomeric ratio, *dr_anti/syn_* from 58:42 up to 99:1) and satisfactory optical purity for the *anti*-isomer (65% < *ee_anti_* < 90%) (Scheme 2A). However, the absolute configuration of the products was not assigned. Higher yields were obtained with electron-withdrawing substituted benzaldehydes compared to those containing electron-donating groups, and reactions with cyclohexanone proceeded with better stereoselectivity (*dr* and *ee*) with respect to cyclopentanone and cycloheptanone [24]. The steric hindrance of the substituent on the aldehyde component exerted some positive influence on the diastereoselectivity, which reached the maximum (*dr* 99:1) with 2,6-dichlorobenzaldehyde. However, no obvious correlation was evidenced for the enantioselectivity and *anti*-aldols from benzaldehyde and both 2-bromo- or 4-bromobenzaldehyde were obtained with the same optical purity (87% *ee*).

Cross-aldol reaction between substituted benzaldehydes and cyclic ketones was also catalyzed by bovine pancreatic lipase (BPL), which has a high degree of homology with PPL, in aqueous buffer and both yield and stereoselectivity were influenced by pH, with optimal conditions observed at pH 5.6. Compared with PPL, BPL was less stereoselective, but excellent yields were obtained with electron-withdrawing substituted benzaldehydes [25] (Scheme 2B). 

In both the above cited reactions (Scheme 2A,B), a negligible amount of products was formed in the absence of the enzymes or with denatured enzymes, while some reactivity, but not stereoselectivity, was observed in the presence of bovine serum albumin. These data support the active role of the used lipases in catalyzing asymmetric aldol condensation.

**Scheme 3:**
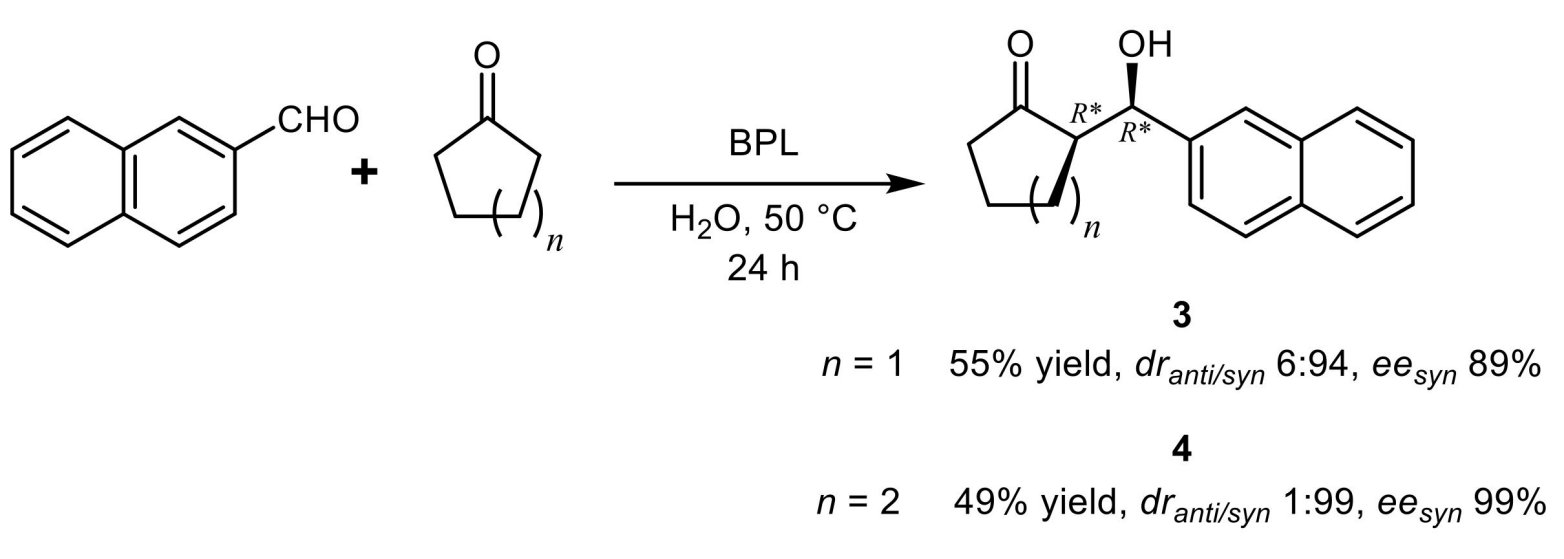
Stereoselective aldol condensation of β-naphtaldehyde and cyclic ketones catalyzed by BPL.

In a subsequent development of the BPL-catalyzed aldol condensation [26], it was found that the use of ILs or DES markedly reduced the product yield, whereas in the presence of water an inversion of the diastereoselectivity was observed from 30 to 50 °C. Considering that the ketone component is in large excess, the reaction actually proceeded in cyclohexanone:water 1:1 mixture and in these conditions the *syn*-isomers of aldols were obtained as main products with moderate diastereo- and enantioselectivity, with the exception of the aldols **3** or **4** deriving from β-naphtaldehyde (Scheme 3).

In the search for other enzymes with catalytic promiscuity, cross-aldol reactions catalyzed by lipase from *Mucor miehei* (MML) have been recently investigated and the reactivity in different reaction media followed the same trends reported for BPL. However, data on diastereoselectivity were not homogeneous and products were not analyzed for their enantiomeric excesses [27]. 

**Scheme 4:**
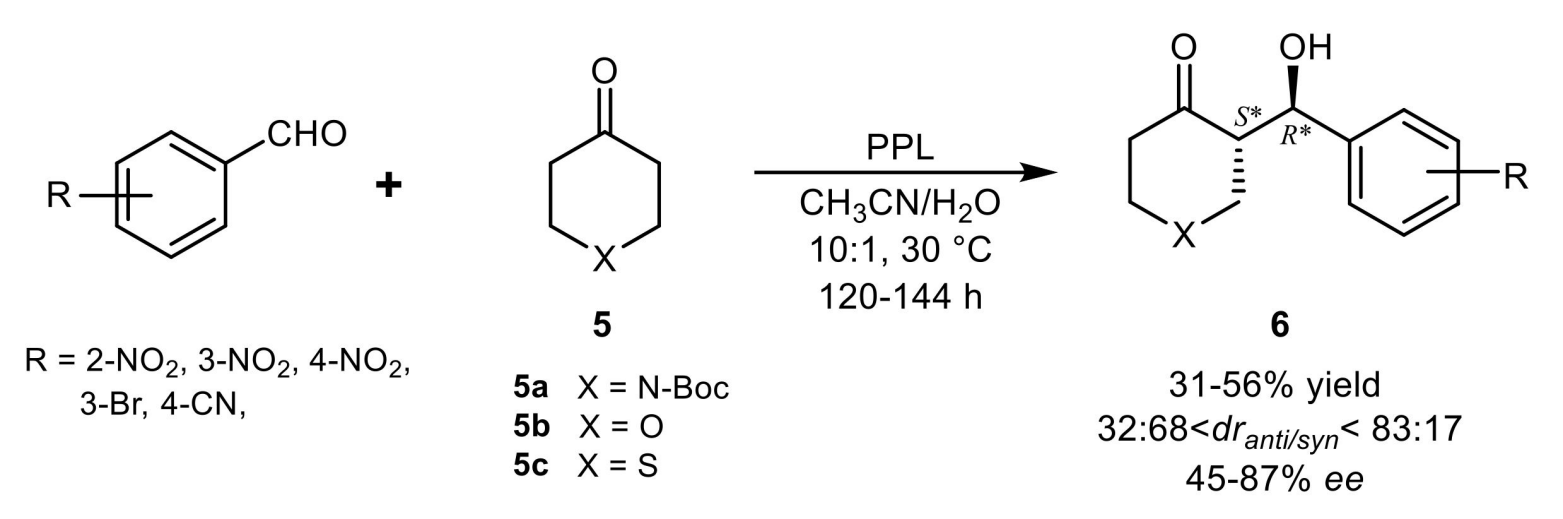
Aldol condensation of aromatic aldehydes and heterocyclic ketones catalyzed by PPL in organic solvent.

In a screening for aldolase activity of some hydrolases in the condensation of *N*-Boc cyclic ketone **5a** and 4-nitrobenzaldehyde, PPL was the most active enzyme and CH_3_CN:H_2_O 10:1 (*v*/*v*) the optimal reaction medium, but lipases from wheat germ and from *Aspergillus niger* also catalyzed the formation of the corresponding aldol product **6**. Although PPL displayed comparable activity in other polar organic solvents, such as THF, DMSO, or DMF (in 10% water mixtures), the highest enantioselectivity in the test reaction was observed in CH_3_CN (59% *ee*) and, interestingly, a reversal of diastereoselectivity was detected in DMSO and DMF. The use of ketone **5b** in CH_3_CN resulted in the preferential formation of *syn* diastereoisomer of **6**, while the highest enantioselectivity with all the ketones **5a**-**5c** was observed in the coupling of 2-NO_2_-benzaldehyde. Although the yields and stereoselectivities were moderately good, even with different substrates (Scheme 4), these aldol reactions evidenced the ability of PPL to display its promiscuous activity in organic solvents as well [28].

**Scheme 5:**
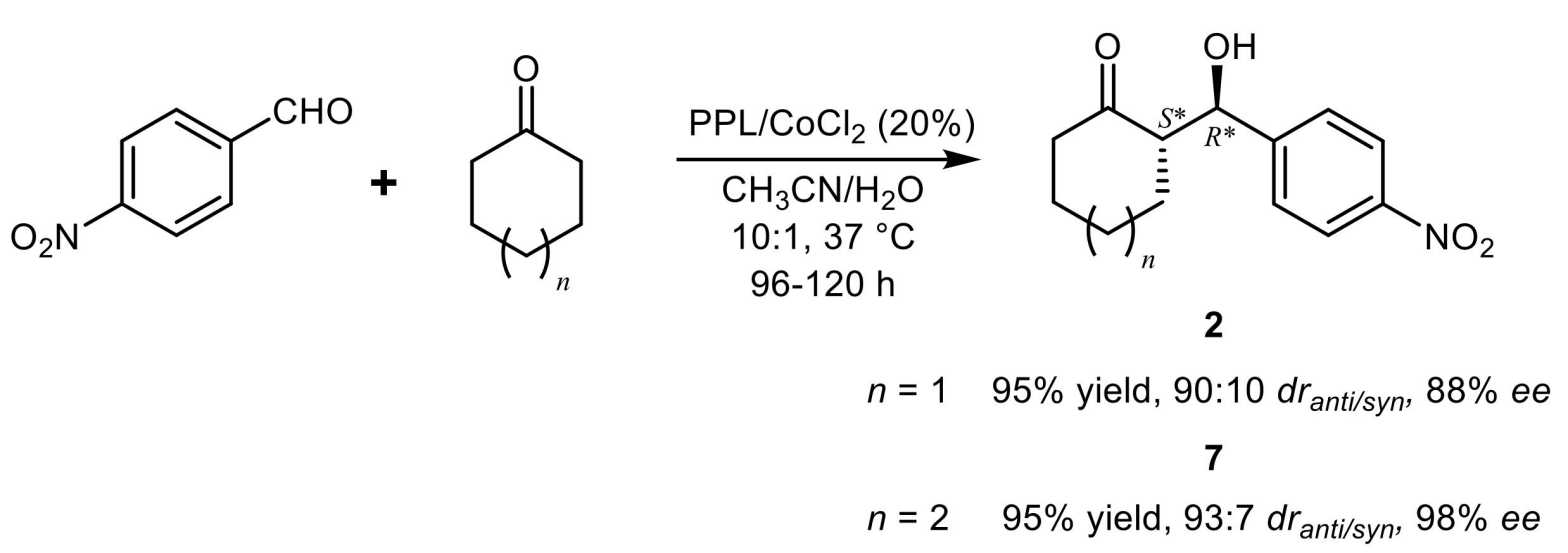
Enzyme/Lewis acid-catalyzed aldol condensation of 4-NO_2_-benzaldehyde and cyclic ketones.

More recently, Yusufoglu and coworkers [29] showed that the CH_3_CN:H_2_O 10:1 (v/v) mixture was the best solvent also for the aldol reactions of cyclohexanone and aromatic aldehydes in the presence of PPL or lipase A from *A. niger* (AL-AN), which displayed comparable activity and stereoselectivity in the model reaction with 4-nitrobenzaldehyde. In the same study, different Lewis acids were also screened as cofactors and CoCl_2_ (20% mol) gave the best results, leading to enhanced product yield and stereoselectivity for both the tested enzymes. In these conditions, even aldehydes with electron-donating substituents gave satisfactory results and high diastereoisomeric ratios (*dr* up to 96:4) and enantiomeric excesses in the range of 70–92% were measured for aldols **2** deriving from the reaction of a variety of aldehyde substrates in the presence of PPL, while AL-AN displayed slightly lower performances. The use of aliphatic ketones led to sensibly lower stereoselectivity, but an excellent result was instead obtained with cycloheptanone (Scheme 5). 

**Scheme 6:**
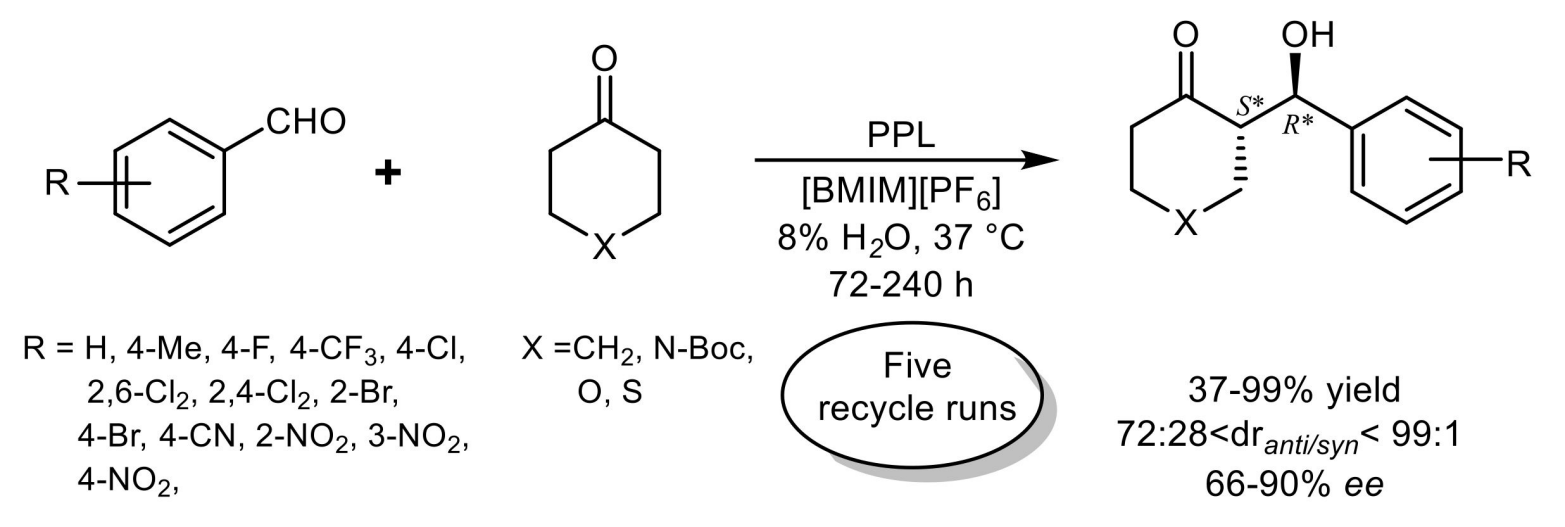
PPL-catalyzed aldol reactions in ionic liquids.

In the first report of an efficient lipase-catalyzed asymmetric cross-aldol reaction in ILs as a more sustainable alternative to organic solvents, only PPL exhibited catalytic activity among seven tested lipases and the presence of water was found essential to achieve good substrates conversion [30]. A series of tetrafluoroborate and hexafluorophosphate salts of imidazolium cations was screened and the use of 1-butyl-3-methylimidazolium hexafluorophosphate [BMIM^+^][PF_6_^–^], with an optimized water content (8%), gave the highest selectivity in the model reaction of 4-nitrobenzaldehyde and cyclohexanone (*dr_anti/syn_* 87:13, 80% *ee*) coupled with 71% product yield. Further refinement of the reaction conditions revealed that yield and diastereoselectivity, but not the enantioselectivity, were affected by the enzyme loading while the raising of temperature above 37 °C led to the erosion of global stereoselectivity. The IL/PPL system displayed excellent recyclability, as no loss in enzyme activity or stereoselectivity was detected over five recycle runs, and broad substrate scope, affording a variety of *anti*-aldols with good to excellent diastereoselectivity (with a maximum value of *dr_anti/syn_* >99:1 for aldol from 2,4-dichlorobenzaldehyde and cyclohexanone) and enantioselectivity (with a maximum of 90% *ee* for aldol from 4-bromobenzaldehyde and cyclohexanone) (Scheme 6). However, pentafluorobenzaldehyde and 2,6-dichlorobenzaldehyde, which reacted with the best diastereoselectivity, gave the aldols with the lowest values of enantiomeric excess, suggesting that the enantioselectivity is governed by factors other than the steric hindrance.

**Scheme 7:**
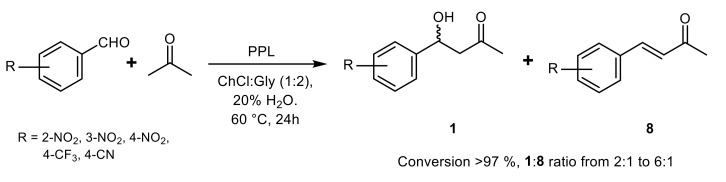
PPL-catalyzed aldol reactions in deep eutectic solvent.

Promiscuous PPL-catalyzed aldol reactions were also reported to proceed in choline-based DES [31] and the concomitant formation of the aldol-dehydration product **8** was observed in the model reaction of 4-nitrobenzaldehyde and acetone at 60 °C (Scheme 7). Experiments without PPL or in the presence of enzyme pretreated with phenylmethanesulfonyl fluoride (an inhibitor of serine hydrolases) supported the occurrence of a specific enzymatic catalysis for both the aldol condensation and the dehydration reactions. 

The enzyme loading, choline chloride:glycerol ratio, amount of water in the reaction medium and substrate concentration were tuned to maximize the **1**:**8** ratio and the optimized conditions were then extended to a series of aromatic aldehydes and cyclic ketones to give the expected aldols in excellent yield and the aldol-dehydration products in a maximum amount of 7%. However, poor diastereoselectivity with a slight preference for the *syn*-isomers (52–66%) of aldols deriving from cyclic ketones was observed and the values of *ee* were not reported for any of the obtained products.

From the above cited papers, PPL emerges as the best lipase for catalyzing asymmetric aldol reactions. However, the lack of information about the absolute configuration of the obtained chiral products is quite surprising and, at the same time, disappointing since these data could be valuable in the definition of the reaction mechanism, which is actually unexplored. 

**Scheme 8:**
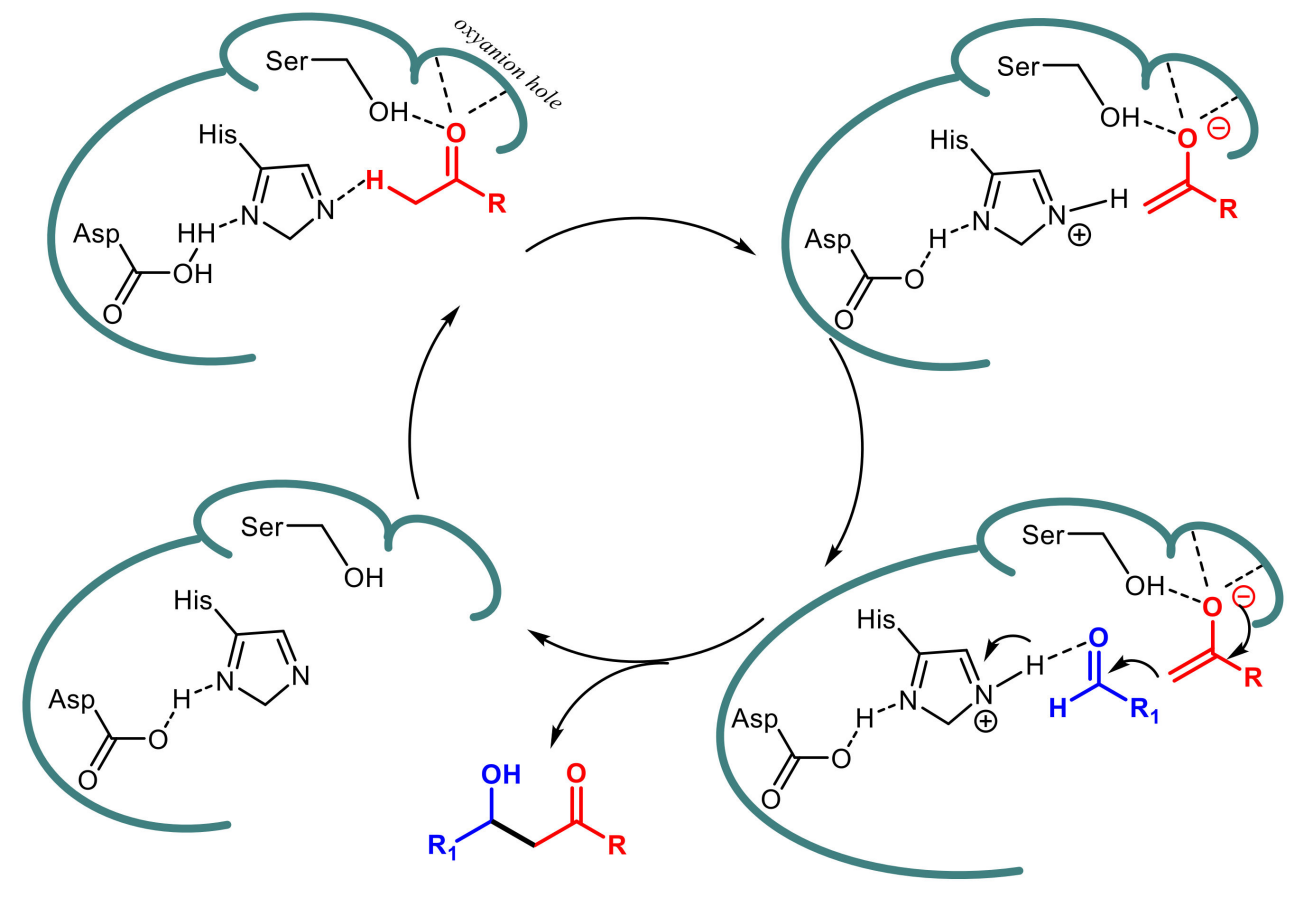
Proposed mechanism for lipase-catalyzed aldol condensation.

Taking into the account the structural features of the active site in serine hydrolases, i.e., the presence of a Ser-Asp-Hys triad and an oxyanion hole, a two-step mechanism has been proposed for lipase-catalyzed aldol reactions [21,23,24,32] and some support comes from ab initio calculations [21] and molecular docking [32]. In the first step the abstraction of an α-proton from the donor component is assisted by Asp-His dyad and leads to an enolate, which is stabilized by a network of hydrogen bonds in the oxyanion hole and/or with Ser residue. Subsequently, a molecule of the acceptor component, held close to the enolate by hydrogen bond with protonated His, can undergo a nucleophilic attack on its carbonyl group to give the aldol product, which is eventually released from the enzyme active site (Scheme 8).

The first step, which resembles the analogous activation of the carbonyl functionality of the substrate by the oxyanion hole in the formation of the acyl-enzyme during the physiological hydrolytic mechanism of lipases, has been predicted to be rate-determining at MP2/6-31+G* calculation level, whereas the B3LYP/6-31+G* values indicated the addition step as crucial for reaction rate. 

**Scheme 9:**
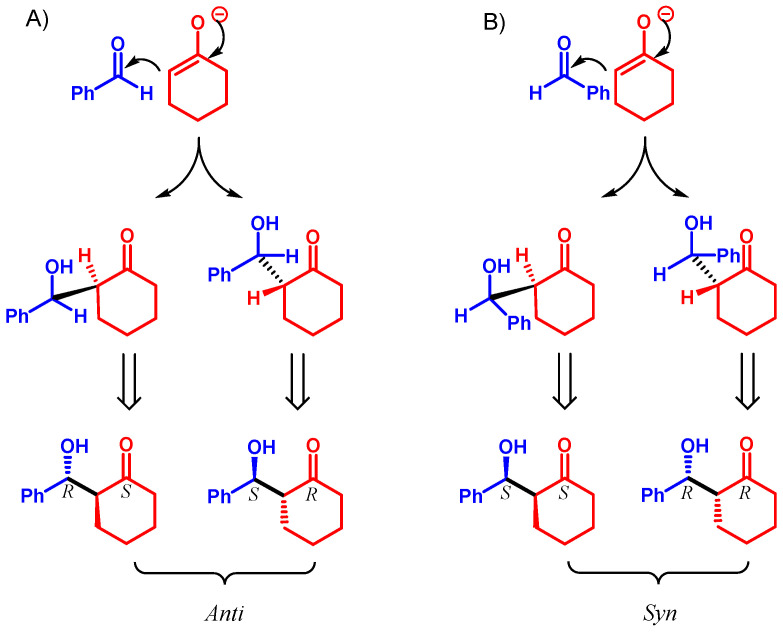
Aldol products deriving from the different relative orientation of the reactants.

The overall stereoselectivity is obviously governed by the relative orientation of the aldehyde acceptor and ketone donor within the active site of the enzyme (Scheme 9) and the marked diastereopreference for the *anti*-isomer, observed in most of the reported lipase-catalyzed aldol reactions between benzaldehydes and cyclohexanone, suggests a preferential orientation of the phenyl group (as in Scheme 9A) in the step of C-C bond formation. However, the higher steric hindrance of naphthyl substituent compared to the phenyl one could force the reactants in the opposite mutual position within the enzyme active site, resulting in the good *syn*-diastereoselectivity reported for products of β-naphthaldeyde and cyclic ketones [26].

## 3. Lipase-Catalyzed Michael Reactions

The 1,4-additions of different nucleophiles to activated olefins are grouped as Michael reactions and represent a versatile method for the construction of C-C or C-X bonds. The olefin component (Michael acceptor) is in most cases an α,β-unsaturated carbonyl compound, but nitroolefins, maleimides, acrylonitrile, and acrylate derivatives also undergo the nucleophilic addition of enolates, amines, thiols, and phosphines (Michael donors) in the presence of a variety of organocatalysts, metal-based or Lewis acid catalysts [33,34].

**Scheme 10:**
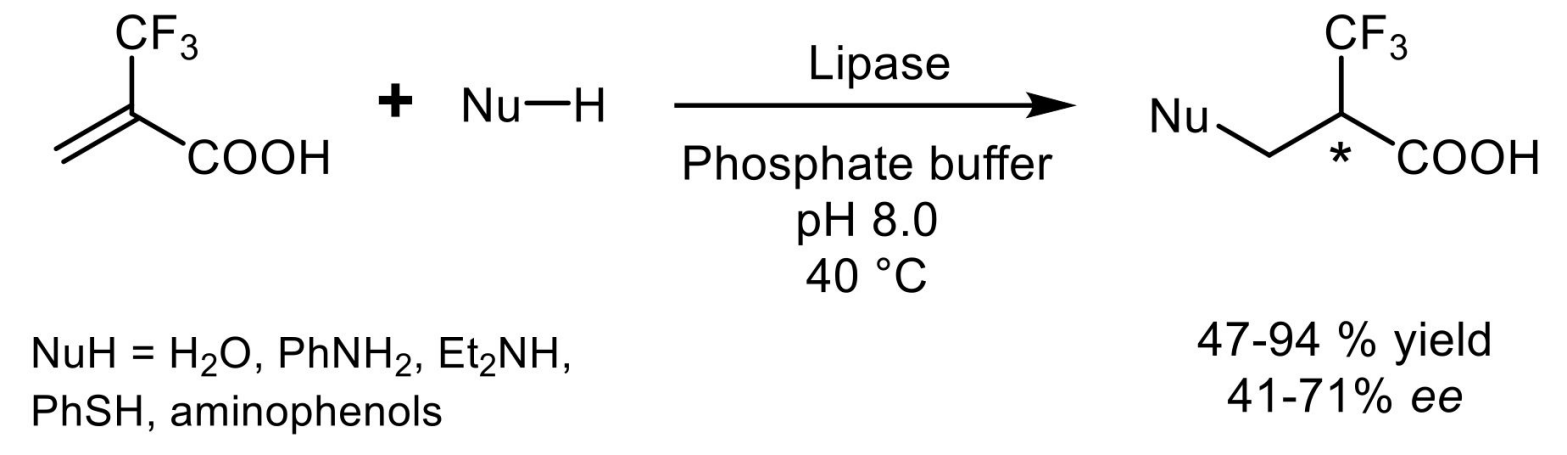
The first reported lipase-catalyzed Michael addition.

In the first report of a lipase-catalyzed Michael reaction, the addition of nucleophiles to 2-(trifluoromethyl)propenoic acid was investigated [20] and lipases from *Candida cylindracea* or *Trichoderma viride* were shown the most effective in catalyzing the addition of water, thiols, and amines, leading the chiral products in moderate to good yield (47–94%) and optical purity (41–71% *ee*) (Scheme 10).

Following this report, the promiscuous catalytic activity of lipases in Michael reactions was mainly focused on the addition of amines or 1,3-dicarbonyl compounds to derivatives of acrylic acid, which gives in most cases achiral or racemic products [35,36,37,38]. Michael additions to *trans*-β-nitrostyrene with different donors in the presence of a variety of lipases have been also explored [39], without any evidence of enantioselectivity. 

**Scheme 11:**
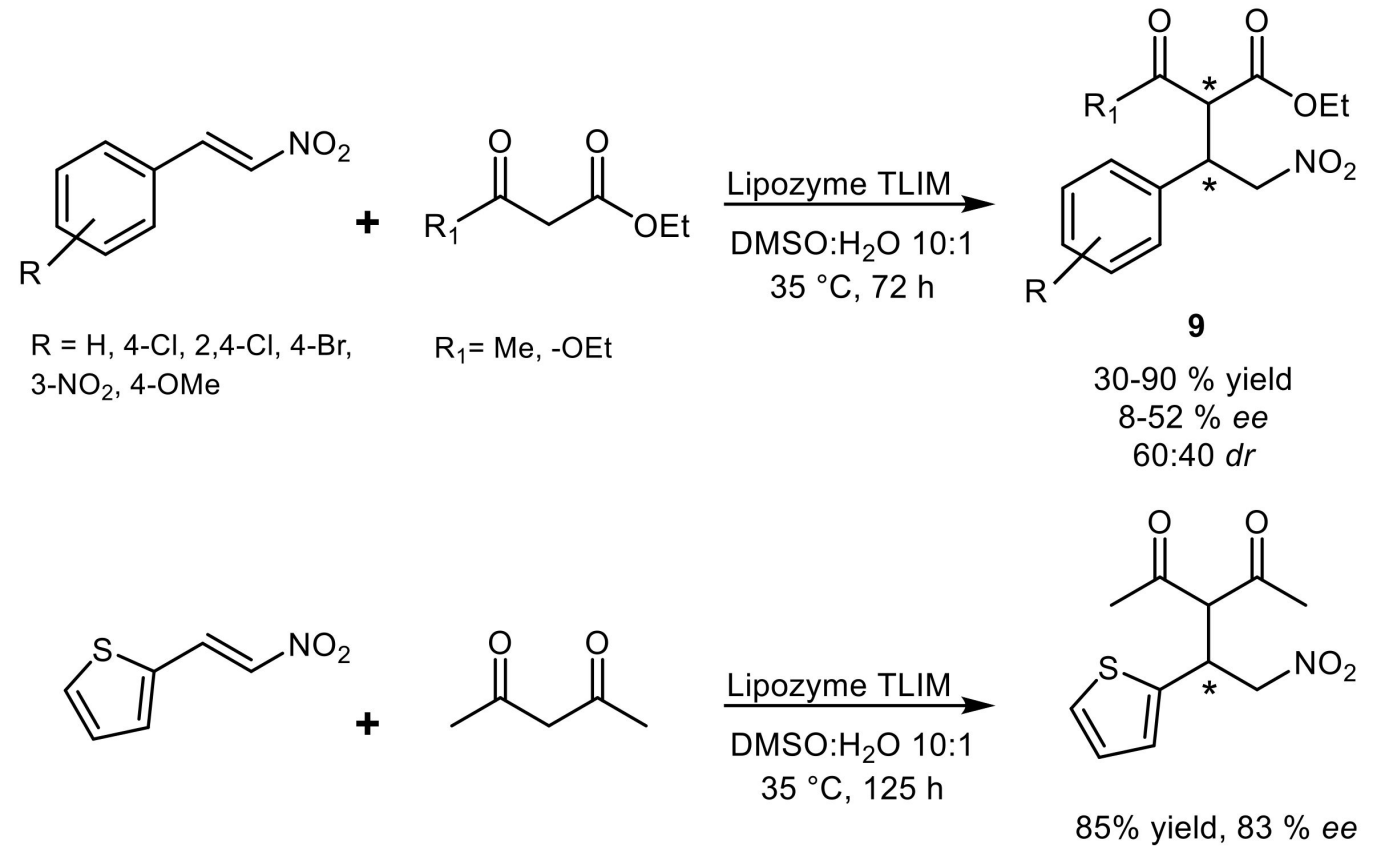
Asymmetric lipase-catalyzed Michael addition of 1,3-dicarbonyl compounds to nitroolefins.

Asymmetric C-C Michael additions between 1,3-dicarbonyl compounds or cyclohexanone and nitrostyrenes were instead reported in the presence of immobilized lipase from *Thermomyces lanuginosus* (Lipozyme TLIM) as biocatalyst in DMSO/H_2_O 10:1. A broad substrate scope coupled with moderate to good yields of products **9** was demonstrated and the best enantioselectivity was observed with thienyl nitroolefin acceptor [40] (Scheme 11). 

**Scheme 12:**
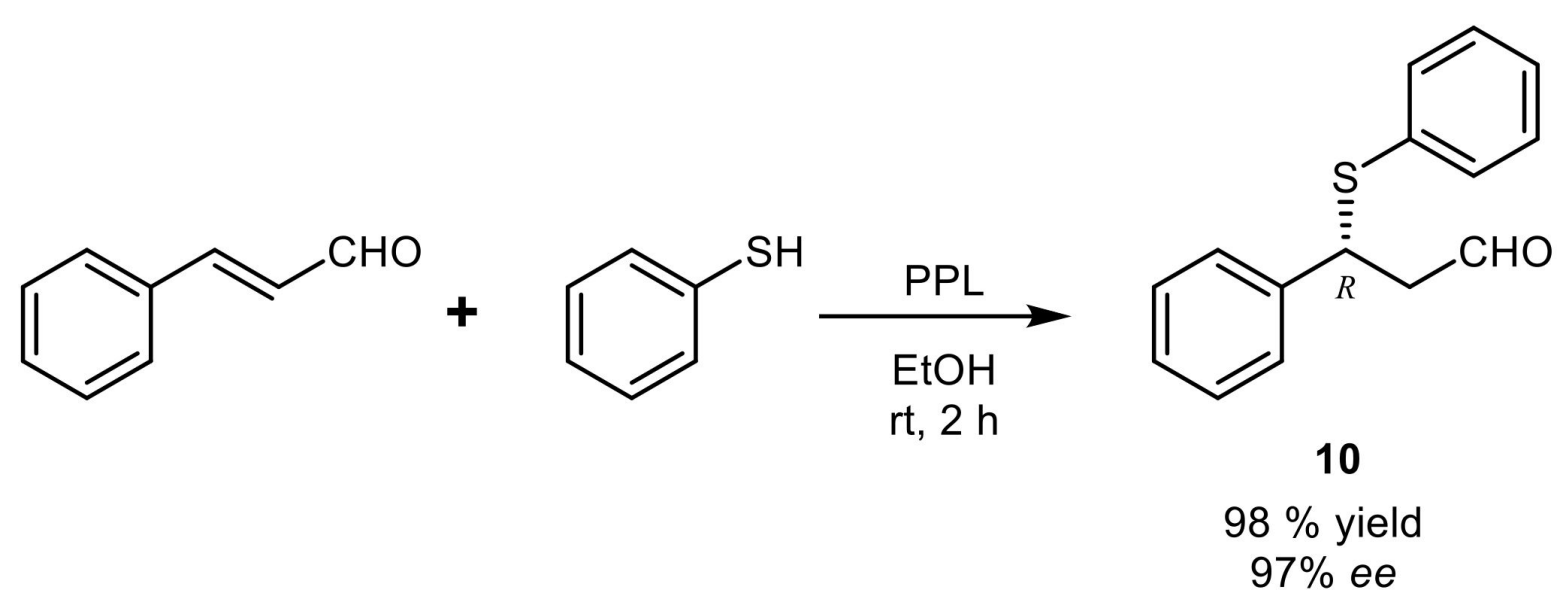
Lipase-catalyzed *thio*-Michael addition of thiophenol to cinnamaldehyde.

An effective protocol for the formation of C-S bond exploited the *thio*-Michael addition of thiophenol to cinnamaldehyde in the presence of lipases [41] and it was optimized for the multi-gram preparation of compound **10**, which was obtained in excellent optical purity and *R*-configuration with PPL in EtOH (Scheme 12). 

**Scheme 13:**
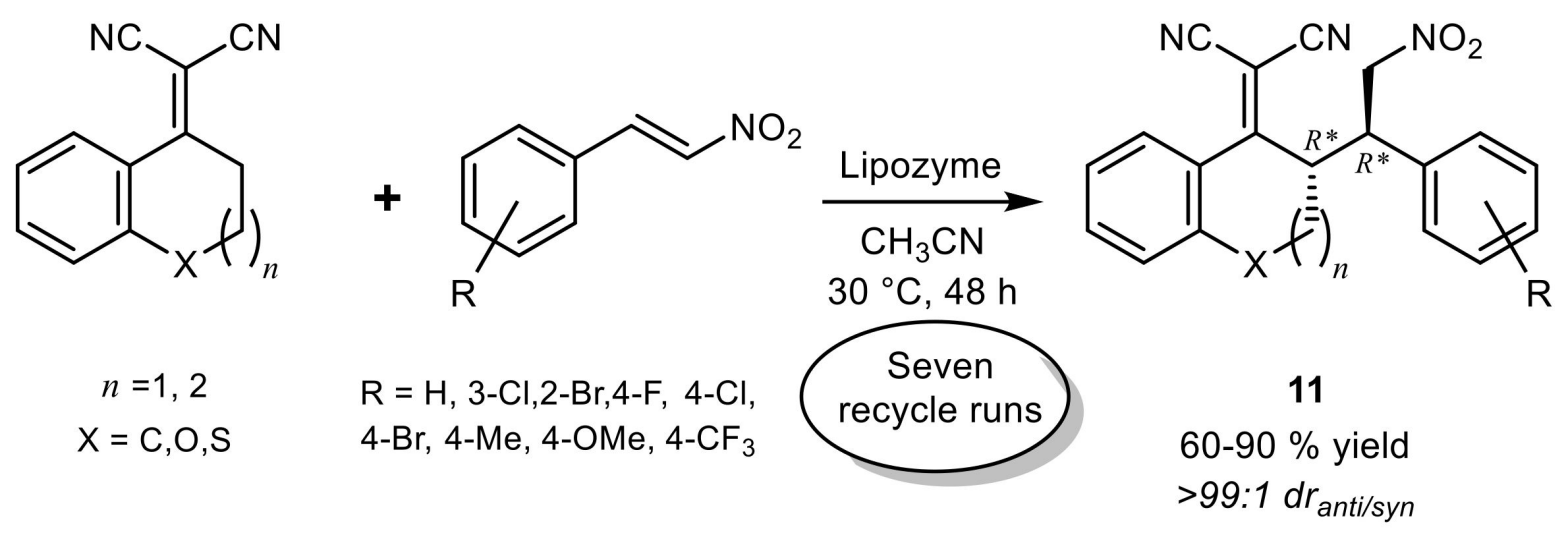
Lipase-catalyzed vinylogous Michael addition.

An immobilized lipase from *Mucor miehei* (Lipozyme) was reported effective in catalyzing the addition of α,α-dicyanoolefins to nitroalkenes with a good substrate scope (17 examples). The addition products **11** were obtained in high yield (60–90%) and excellent diastereoselectivity (*dr_anti/syn_* >99:1), but their optical purity was not determined (Scheme 13). It was shown that lipase is essential for this vinylogous Michael addition, and the enzyme was recycled over seven consecutive runs with very slight loss of activity [42]. 

**Scheme 14:**
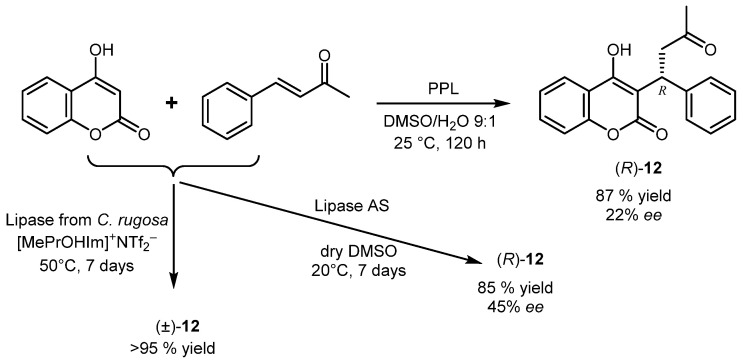
Synthesis of warfarin by lipase-catalyzed Michael addition in different reaction conditions.

Some interest has been focused on the synthesis of warfarin **12** through a biocatalyzed Michael addition of 4-hydroxycoumarin to benzylideneacetone in wet [43] or anhydrous DMSO [44] in the presence of different lipases, but despite the obtained yield of the target drug (85–87%) being satisfactory, the enantioselectivity was quite low (Scheme 14).

More recently, the use of ionic liquids as reaction medium was shown feasible for this reaction, but detrimental for the enzyme enantioselectivity, leading to racemic warfarin in >95% yield [45].

**Scheme 15:**
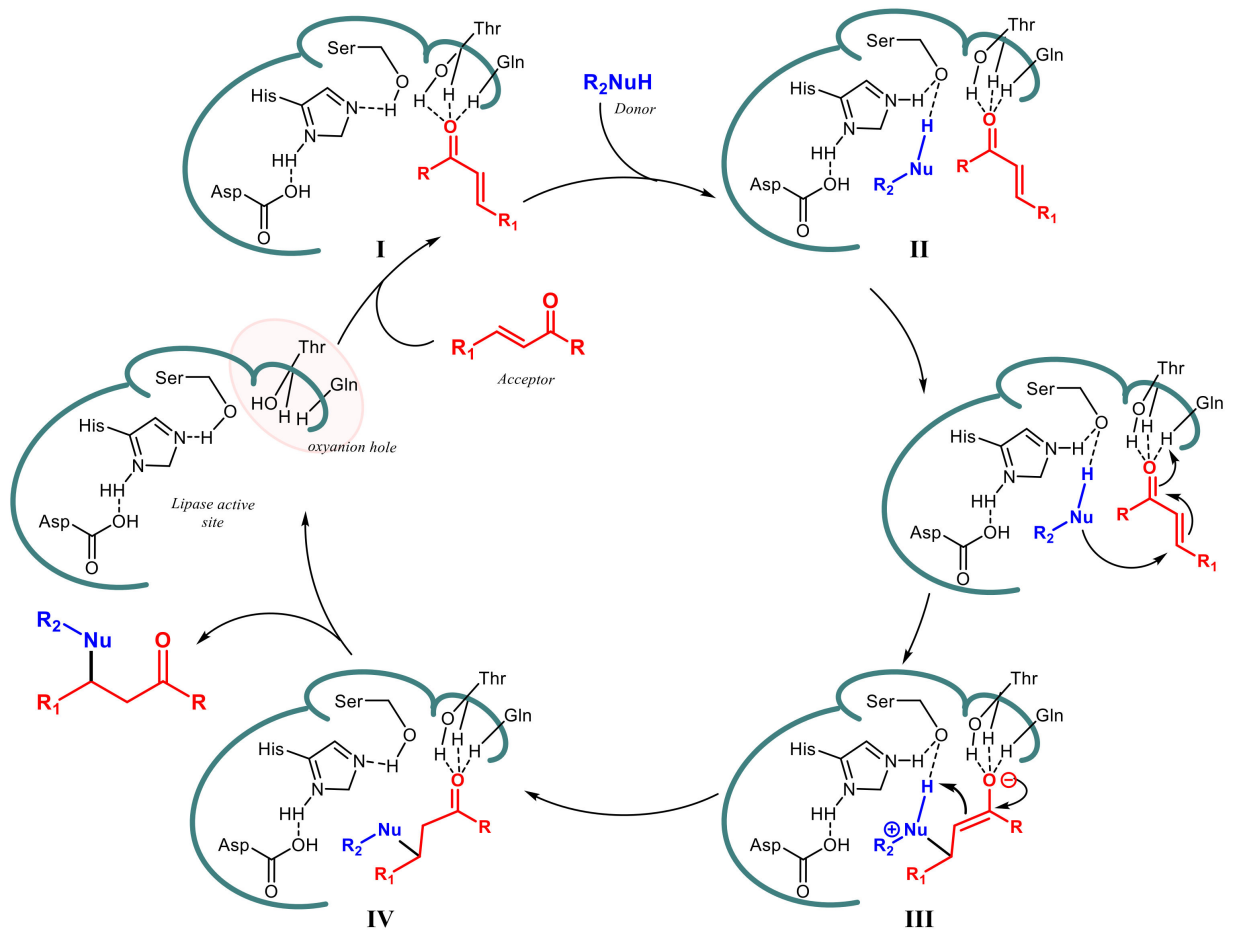
Proposed mechanism for lipase-catalyzed Michael addition.

Regarding the mechanism of these biocatalyzed addition reactions, it has been proposed [39,46,47] that the Michael acceptor is activated by means an array of hydrogen bonds between its polar group (-COR, -NO_2_, -CN) and amino acid residues (Thr40 and Gln106) into the so called “oxyanion hole” in the active site of lipase (I in Scheme 15). In this way, the molecule is accommodated in the catalytic site and increases its electrophilic character. A hydrogen bond between Ser105 and the nucleophile (-NH, -SH, carbanion), assisted by His224, facilitates the anchoring of the donor component in the active site (II) followed by the formation of a new covalent bond through a zwitterionic intermediate (III) which can be stabilized by both the oxyanion hole and Ser-His-Asp triad. After the interconversion of the enol intermediate into the keto form, the product is then released (IV) and the active site of the enzyme restored to the initial state (Scheme 15). 

This mechanism proposal was supported by calculation on model reactions and by experiments with mutants of *Candida antarctica* lipase B (CAL-B) lacking Ser105 or His224 that evidenced a crucial role of these amino acid residues in the transfer of a proton to the product [46,47]. On the other hand, docking studies on the (*R*)- and (*S*)-enantiomers of the Michael adduct from 3-buten-2-one and methyl 2-nitropropanoate did not reveal differential binding modes, in agreement with the experimental lack of enantioselectivity [39].

## 4. Lipase-Catalyzed Multicomponent Reactions

Multicomponent reactions (MCRs) are attracting great interest as a powerful synthetic tool for the construction of multiple C-C (or C-X) bonds in a single step, without the need to isolate and purify intermediates, thus addressing the demand of more sustainable processes. For their low substrate specificity and “promiscuous activity”, lipases could be an ideal biocatalyst for MCRs, which often imply sequences of aldol condensation/Michael addition reactions.

**Scheme 16:**
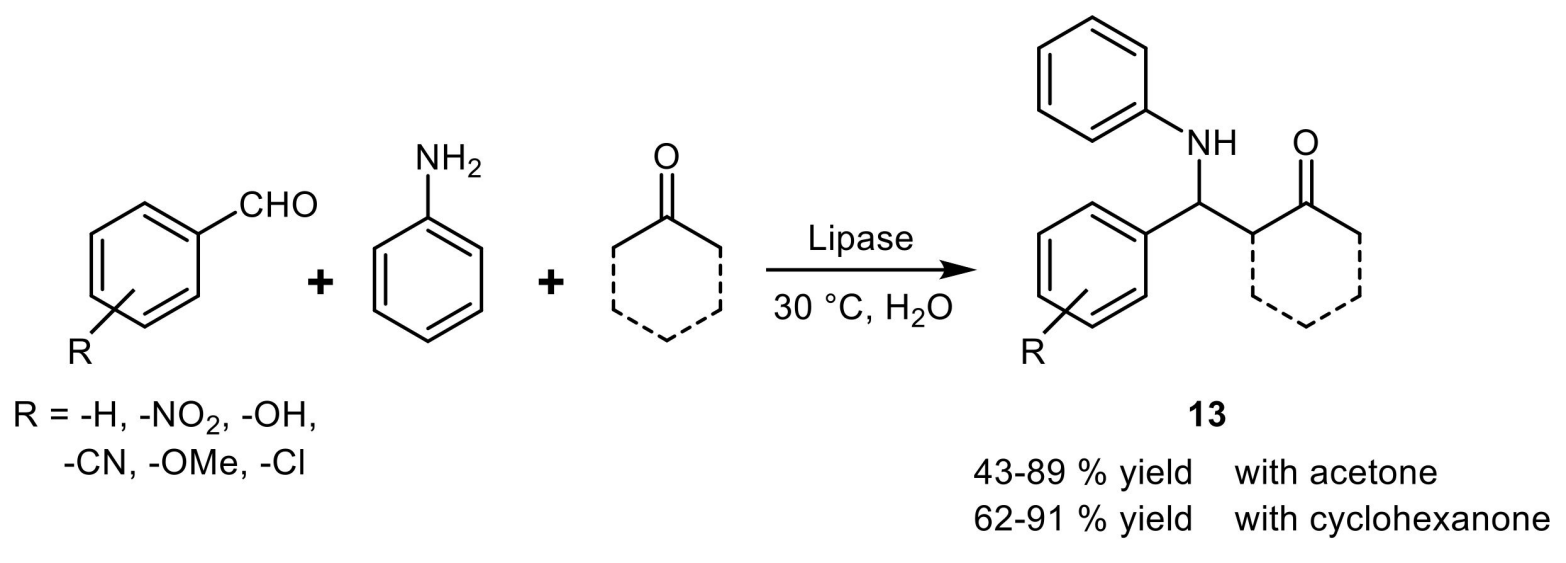
Lipase-catalyzed Mannich reaction.

In the first example of lipase-catalyzed Mannich reaction, a well-known three-component condensation, the highest yields (43–89%) of the target amines **13** were obtained with MML and the reaction rate was found strongly accelerated by the presence of water in the organic solvent with an optimal 40–50% content (v/v) [48]. Although the reaction scope was further extended to some ketones other than acetone by using lipase from *Candida rugosa* (CRL) [49], all the products were obtained with low diastereoselectivity (when applicable) and in racemic form (Scheme 16). 

**Scheme 17:**
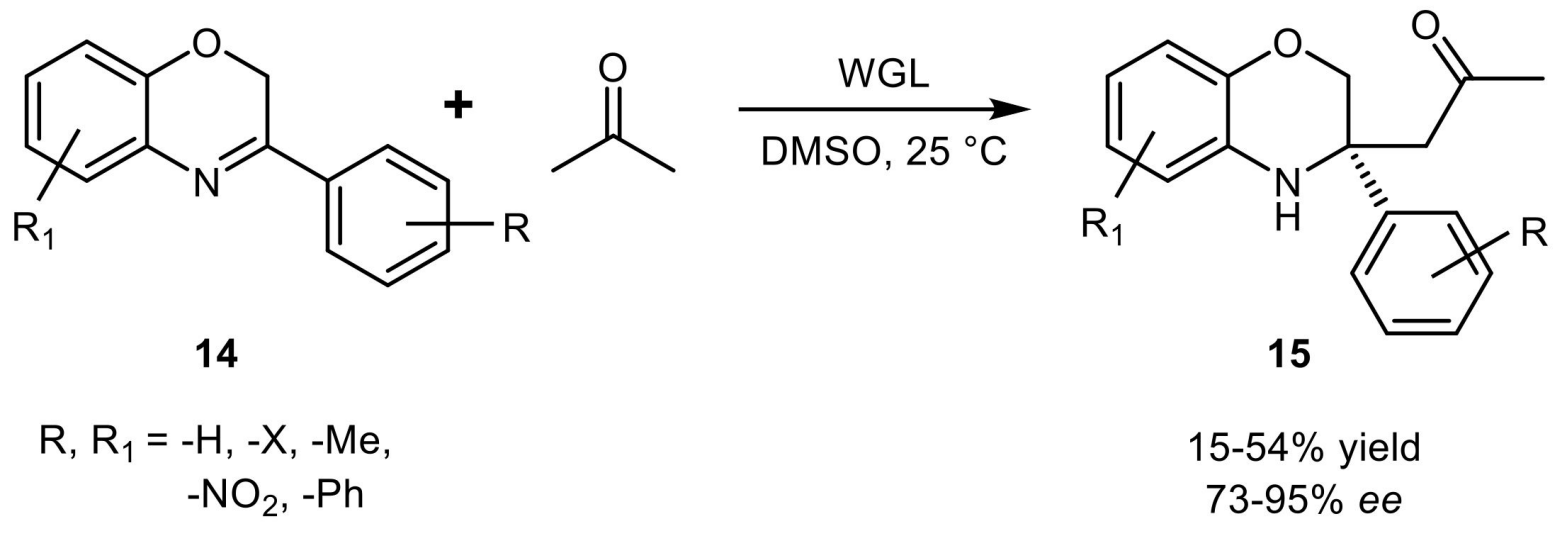
Asymmetric Mannich reaction catalyzed by wheat-germ lipase.

A remarkable enantioselectivity was instead achieved in the synthesis of amines **15** by the Mannich reaction of preformed ketimines **14** with acetone in the presence of wheat germ lipase (WGL), a plant enzyme which has been less investigated compared to the other lipases from microbial source (Scheme 17). Although the product yields varied from low to moderate, the optical purity was less influenced by the reaction conditions and compounds **15** were obtained with averaged 87% *ee* [50].

**Scheme 18:**
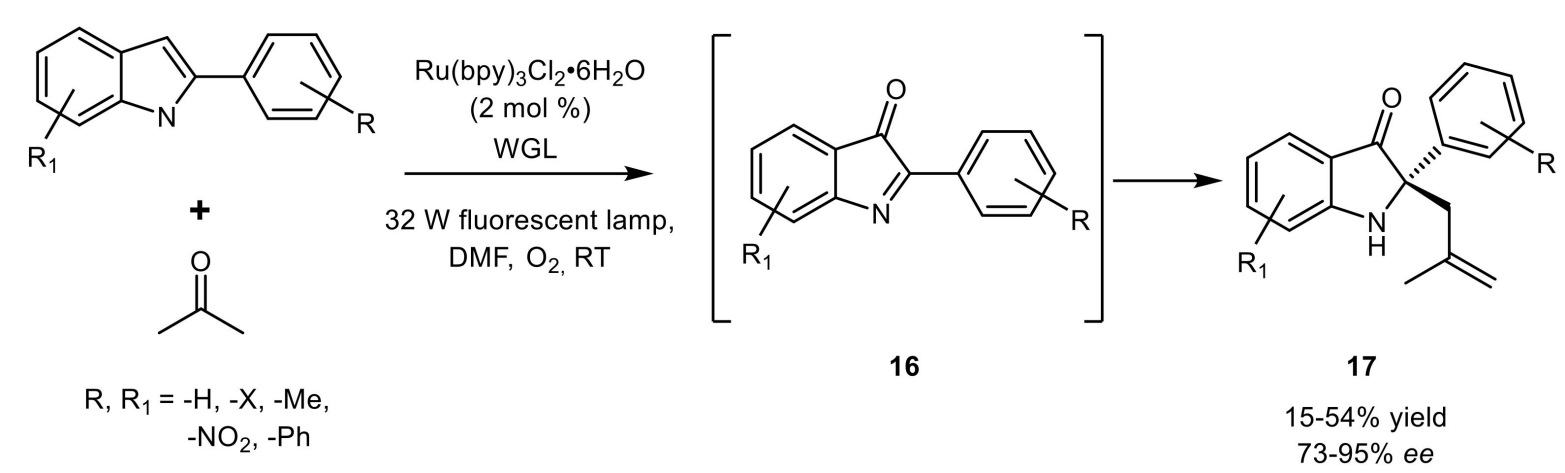
Combined photocatalysis and biocatalysis for the synthesis of 2-fully substituted indoles.

This promiscuous activity of WGL was also exploited for the synthesis of 2,2-disubstituted indol-3-ones in a one-pot combination with the photoredox catalyst Ru(bpy)_3_Cl_2_, which is able to oxidize 2-arylindoles in the presence of oxygen under visible-light irradiation, thus generating in situ the reactive ketimines **16**, then alkylated by acetone (Scheme 18).

The photocatalytic and biocatalytic concurrent reactions were effective in giving the target products **17** with good optical purity and moderate yields, also showing a valuable substrate tolerance as explored with differently substituted 2-arylindoles and with ketones other than acetone [51]. Notably, both the above reactions catalyzed by WGL offer a valuable approach for access to heterocycles bearing a quaternary stereocentre, whose enantioselective synthesis is often challenging. 

**Scheme 19:**
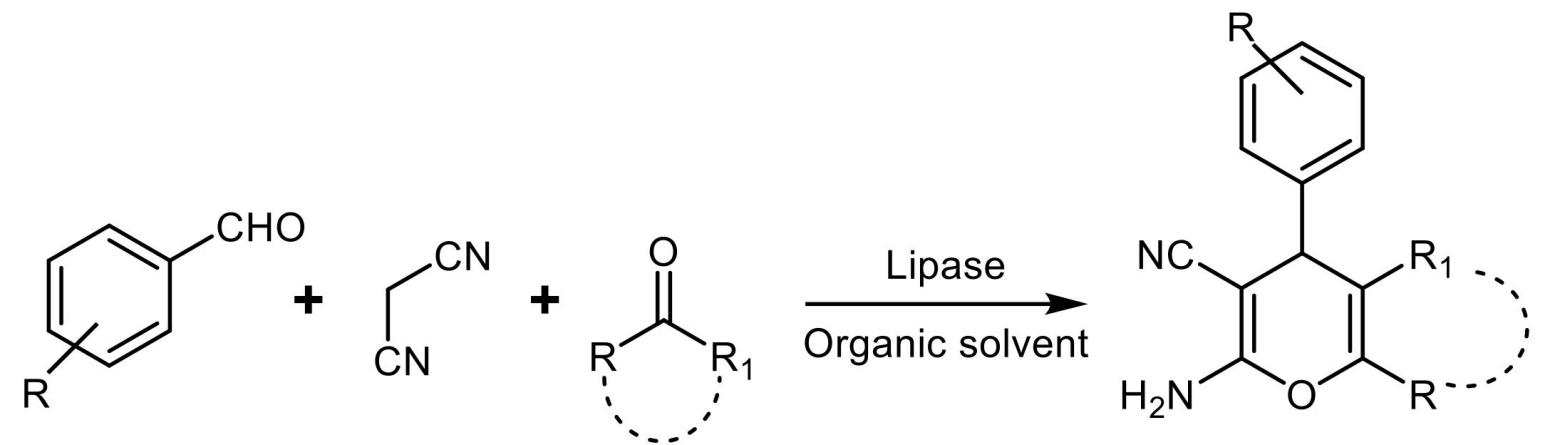
Lipase-catalyzed multicomponent one-pot synthesis of tetrahydropyranes.

Although several lipases are able to catalyze MCRs of malononitrile, aldehydes, and active carbonyl-containing compounds and this process has been applied to the one-pot synthesis of dihydropyran and chromene derivatives (Scheme 19), in most cases with excellent product yield and good functional group tolerance, no enantioselectivity was detected in all the cases [52,53,54,55,56]. 

**Scheme 20:**
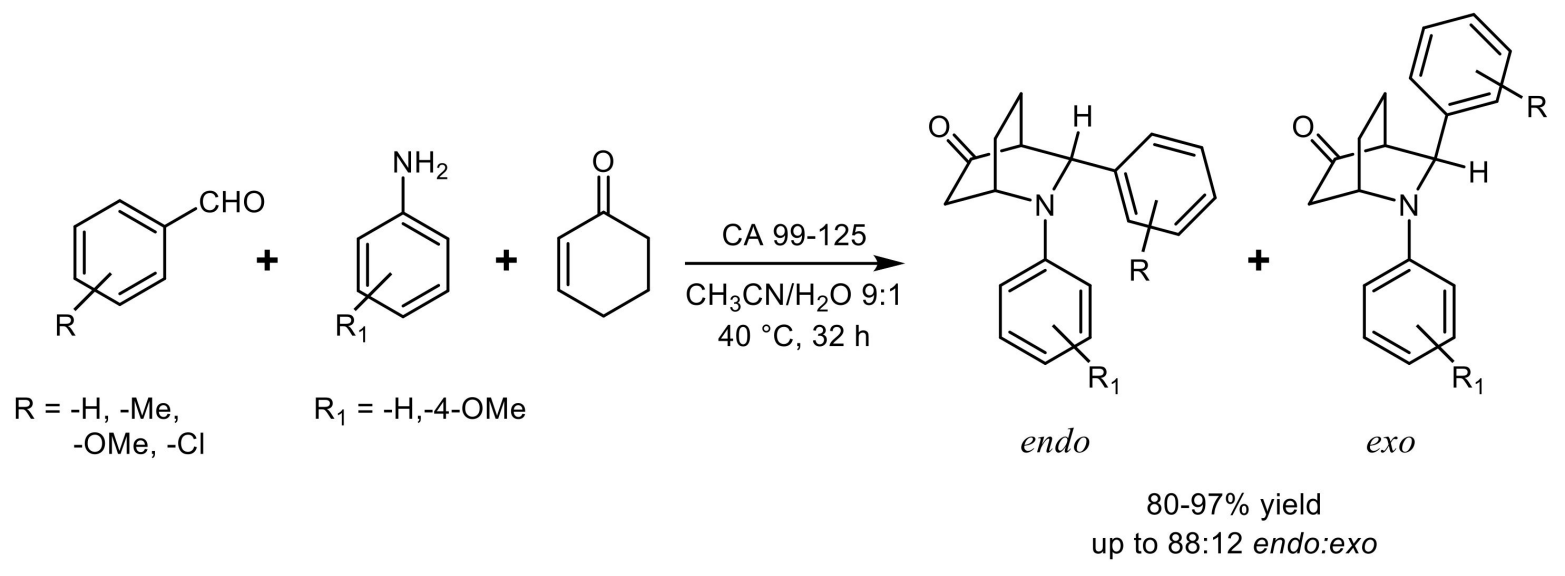
Diastereoselective lipase-catalyzed Diels-Alder reaction.

Lipase from *Candida Antarctica* sp. 99–125 proved to be a good catalyst for the direct three-component *aza* Diels-Alder reaction between aromatic aldehydes and anilines with 2-cyclohexen-1-one and the best *endo*/*exo* diastereoselectivity (95:5) was observed in DMF or DMSO, while the highest product yield was obtained in CH_3_CN. Both reactivity and stereoselectivity were affected by the amount of water and a good compromise was found by using CH_3_CN:H_2_O 9:1 as reaction solvent (Scheme 20) [57]. 

**Scheme 21:**
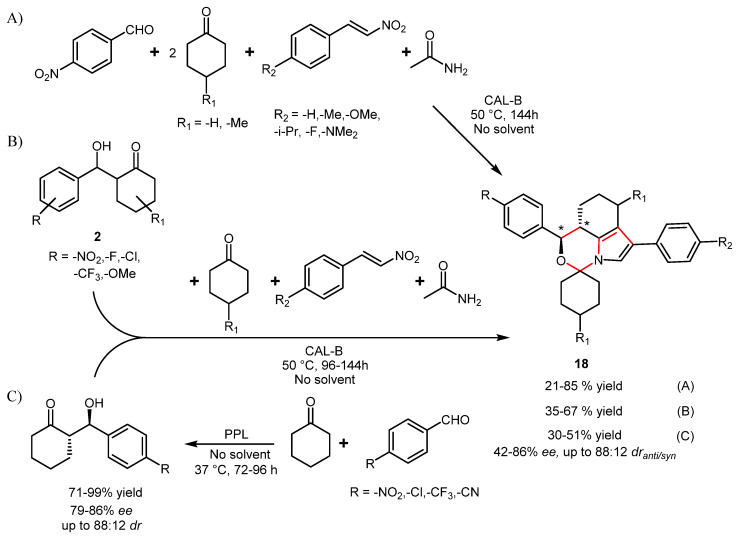
Alternative routes for the synthesis of spirooxazines catalyzed by lipase.

The construction of heterocyclic spiro skeleton, which is synthetically demanding and often achieved through a sequence of different condensation reactions, was successfully achieved by one-pot four-component reaction in the presence of CAL-B (Scheme 21, route A) and some control experiments confirmed the active role of the enzyme in the MCR process. It was also shown that acetamide acted as co-catalyst in promoting the aldol condensation, which is the first step in the reaction cascade, as well as in providing the source of N atom in the final products [58]. The process was very effective in providing the simultaneous construction of six C-C/N bonds and two rings in a single step, but the lack of stereoselectivity of CAL-B in catalyzing the aldol condensation, where the stereocenters of the final spiro-compounds are generated, resulted in racemic products.

In a subsequent optimization of the process (Scheme 21, route B), the use of a preformed aldol intermediate **2** (even in optically pure form) as substrate was proposed and the reaction scope fully explored [59]. The feasibility of a two-lipase telescopic reaction based on PPL-catalyzed aldol condensation of aldehydes with cyclohexanone followed by removal of the enzyme by filtration and simultaneous addition of the other reagents and CAL-B was also investigated and by this route a series of chiral spirooxazines **18** with up to 86% *ee* and 83:17 *dr* was obtained (Scheme 21, route C) [60]. 

The putative mechanisms proposed for these lipase-catalyzed MCRs include common steps: (a) the activation of the carbonyl component by serine residue in the oxyanion hole through the formation of the corresponding enolate and (b) the formation of a Schiff-base assisted by His-Asp dyad in the catalytic site of lipase. The formed intermediates then react via a Michael/Mannich process and the final product is released while the catalytic cycle is restored.

## 5. Conclusions

The study of the promiscuous activity of lipases is still in its infancy, but interesting potential has emerged for its application in organic synthesis, especially for multicomponent reactions in which multiple bonds and functional groups can be established in one step. Although in many cases the formation of new C-C bond(s) is accompanied by the generation of new stereogenic centre(s) in the product molecule, the stereoselectivity of the employed enzymes is still not rationalized. Studies aimed at monitoring the variation of stereoselectivity in function of the substrate or the experimental conditions are lacking, and the absolute configuration of the products has been assigned only in rare cases. Computational approaches, such as ab-initio protein folding, molecular docking, and molecular dynamics, could provide useful information for understanding the origin of stereoselectivity (or lack thereof) and supporting the engineering of the active site of the enzyme [61]. The use of the modern techniques of directed evolution appear even more promising in obtaining engineered biocatalysts with enhanced stereoselectivity and substrate scope [62]. 

The optimization of lipase-catalyzed promiscuous reactions has been mainly focused on the product yield, but the expanded application of these biocatalyzed processes even in asymmetric synthesis would be favorably accepted, offering an alternative to the use of metal-based catalysts or organocatalysts.

## Data Availability

Not applicable.
